# Targeting Inflammation in the Diagnosis, Management, and Prevention of Cardiovascular Diseases

**DOI:** 10.5334/gh.1156

**Published:** 2022-11-02

**Authors:** Akira Matsumori

**Affiliations:** 1 Clinical Research Institute, National Hospital Organization, Kyoto Medical Center, 1–1 Fukakusa Mukaihata-cho, Fushimi-ku, Kyoto 612–8555, Japan

**Keywords:** biomarkers, cardiovascular diseases, immunoglobulin light chains, inflammation, nuclear factor kappa B, Pycnogenol

## Abstract

Inflammation plays an important role in the development and progression of cardiovascular diseases (CVDs). Hypertension and hyperlipidemia are the key risk factors of CVDs and induce inflammation in the heart and vessels. Unhealthy diet, infection, and smoking coupled with genetic factors lead to the development of CVDs as well as induce inflammation. Risk factors of CVDs such as hypertension and hyperlipidemia along with diabetes activate nuclear factor kappa B, which regulates the transcription of immunoglobulin free light chain (FLC) κ in B cells and the production of multiple inflammatory molecules, leading to inflammation. FLCs are novel biomarkers of inflammation, and their levels have been associated with overall mortality in a general population and with cardiovascular outcomes. In this review, the role of inflammation in the pathogenesis of CVDs, new biomarkers of inflammation, and dietary options counterbalancing inflammatory processes, such as the polyphenol-rich French maritime pine bark extract Pycnogenol, are discussed.

## 1. Introduction

Today, 71% of all deaths globally are a result of noncommunicable diseases (NCDs). As the name indicates, NCDs are not transmissible by pathogens but develop as a result of the combinations of behavioral and environmental factors impacting genetic expression and physiological health. Four groups of diseases are attributed to causing 80% of all premature NCD-related deaths: diabetes, chronic respiratory disease, cancer, and cardiovascular diseases (CVDs) are considered the most prevalent NCDs. On an annual basis, diabetes accounts for 1.5 million deaths followed in increasing order by respiratory diseases (4.1 million deaths), and cancer (9.3 million deaths), leaving CVDs as the most abundant cause of NCD-related death with 17.9 million. Behavioral factors such as unhealthy diets, physical inactivity, tobacco use, and excessive use of alcohol all increase the risk of death from NCDs [1]. Although unhealthy diets, smoking, scant exercise, and excessive alcohol intake are well-known risk factors of CVDs, the precise pathogenetic mechanisms leading to CVDs remain to be fully uncovered. Recently, inflammation has been shown to play an important role in the progression of CVDs, and most of the risk factors induce cellular stress and inflammation [2]. In this review, the role of inflammation in the pathogenesis of CVDs, new biomarkers of inflammation, and dietary options counterbalancing inflammatory processes, such as the polyphenol-rich French maritime pine bark extract Pycnogenol^®^ (PYC), are discussed.

## 2. Role of inflammation in the pathogenesis of CVDs

### 2.1. Smoking

Smoking is responsible for many diseases, and its role in cardiovascular diseases has been well proven since it promotes free radical formation, endothelial dysfunction, and inflammation. It was shown to induce the production of multiple pro-inflammatory cytokines while decreasing the levels of anti-inflammatory cytokines. Smoking has also been shown to activate macrophage and dendritic cell activity [3,4].

### 2.2. Diet

Clinical investigations of dietary patterns have shown a relationship between the diet and inflammation [5]. A comprehensive evaluation of the diet examining multiple foods as well as nutrient intakes can be useful in establishing dietary patterns [6]. Recently, published evidence shows the potential impact on cardiovascular disease risk of unhealthy dietary patterns and specific foods [7]. Results of a study that followed 166,000 women and 44,000 men over 24 to 30 years were recently reported by Li and coworkers. The results of the investigation pointed to an increased risk of CVDs being associated with the inflammatory potential of the diet, which was based on a predefined dietary inflammatory pattern score. A higher dietary inflammatory index score positively correlated with a higher risk of CVDs with incidence of stroke reported as high as 28% along with 46% for ischemic heart disease [8]. The results of this long-term, follow-up study provide clear evidence that pro-inflammatory dietary habits are linked to an increased risk of developing CVDs [7].

### 2.3. Atherosclerosis

**Figure 1:**
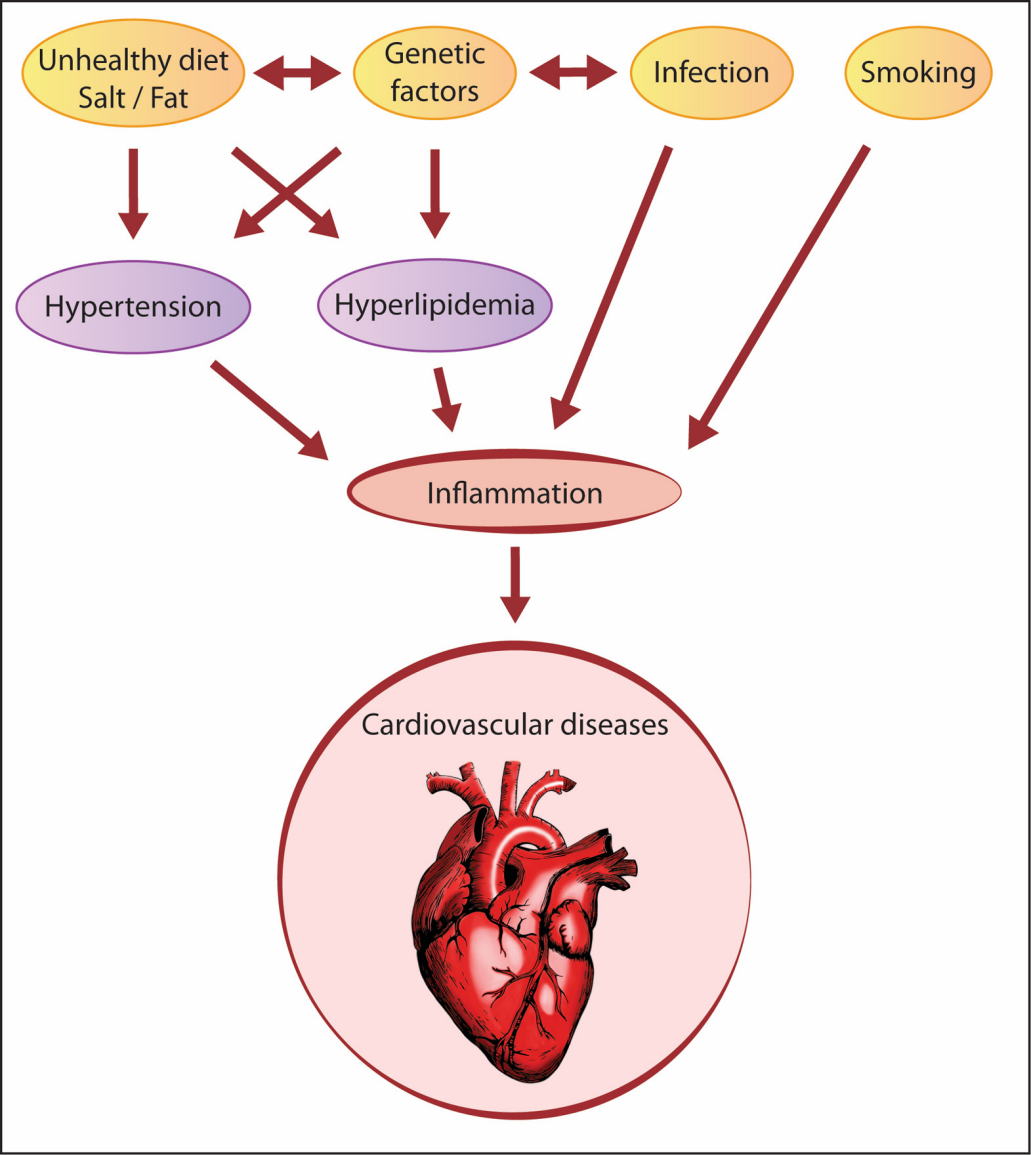
Risk factors of cardiovascular diseases (CVDs) and inflammation. Hypertension and hyperlipidemia are the risk factors of CVDs and induce inflammation in the heart and vessels. Unhealthy diet along with genetic factors lead to the development of hypertension and hyperlipidemia and induce inflammation. Smoking and infection also induce inflammation and lead to CVDs.

Hypertension, hyperlipidemia, and atherosclerosis are the risk factors of CVDs and induce inflammation in the heart and vessels (Figure 1). The most common contributing factor to the development of coronary heart disease is atherosclerosis. It is now largely accepted in the literature that atherosclerosis as a condition marked by chronic, low-grade inflammation. Investigations into the pathophysiology of atherosclerosis and plaque vulnerability have indicated a number of inflammatory mediators that act as causal agents and show potential use as surrogate biomarkers [9,10,11]. Building on the background of traditional medicinal therapies, the observations from epidemiological studies showing that inflammatory biomarkers are associated with CVD risk support the theory that targeted anti-inflammatory therapies may be a promising strategy to lower long-term cardiovascular disease risk. Pharmaceuticals commonly used in the treatment of CVDs, such as statins, have been shown in a large number of randomized controlled trials to have an anti-inflammatory effect, which may explain their efficacy in the prevention of primary and secondary cardiovascular events. Additionally, multiple anti-inflammatory pharmaceuticals are also being investigated for their potential use in lowering cardiovascular disease risk related to atherosclerosis [12]. While controlled clinical studies with antibiotics have not shown to reduce the recurrence of cardiovascular events and vaccination strategies targeted at lowering CVD risk have yet to prove effective in clinical use [13], evidence supporting the use of anti-inflammatory therapies, including anticytokine treatments [14,15] and colchicine [16,17], is beginning to demonstrate efficacy. This growing evidence linking inflammation and related immune mechanisms to both emerging as well as traditional risk factors of CVDs and atherosclerosis may offer novel avenues for intervention [18].

### 2.4. Hypertension

The most common risk factor associated with heart failure is hypertension. Hypertension accounts for approximately 25% of heart failure. Hypertensive heart disease, which manifests clinically as diastolic or systolic heart failure, is marked by cardiac hypertrophy and fibrosis. The development of hypertension is linked to inflammation from immune cells accumulating in the vasculature and impairing blood vessel function [19]. An animal model study involving Dahl salt-sensitive hypertensive rats fed with a high-salt diet showed an increased expression of interleukin (IL)-1β in the heart associated with the rapid development of left ventricular hypertrophy and impaired myocardial function with chamber dilation. The increased expression of IL-1β was observed to further continue to increase with the development of heart failure in this study [20,21]. Additionally, the number of macrophages in the interstitium increased along with monocyte chemoattractant protein-1 (MCP-1) expression. Recent research suggests that macrophages activated by chemokines are acting as important sources of increased circulating pro-inflammatory cytokines. A separate study showed that cyclic mechanical stretch applied to endothelial cells significantly increased the concentration of MCP-1 [22]. Such findings suggest that mechanical stretch may induce genetic expression of chemotactic factors for macrophages, which are noted to be a main source of cytokines, including IL-1β [23]. A recent meta-analysis of human trials with heart failure patients also confirmed that immunomodulation, e.g., with anticytokine therapy, improved cardiac structure and function [24].

### 2.5. Ischemic heart disease

The hallmark of ischemic heart disease is a thrombotic occlusion of a coronary vessel as a result of atherogenesis. The occlusive event is followed by immune cell recruitment to the vessel wall and myocardial infarction [25]. Plaque rupture can result in ischemic death of cardiomyocytes, which are replaced by scar tissue. Inflammation and immune cell infiltration in response to cardiomyocyte necrotic death are critical for cardiac repair after myocardial infarction. Optimal healing includes an inflammatory and reparative phase yielding a stable scar. In contrast, excessive inflammation and fibrosis promote cardiac dysfunction and risk of arrhythmia [26]. A rat model of myocardial infarction investigated the role of inflammatory mediators. The study showed that gene expression of IL-1β, IL-6, and tumor necrosis factor (TNF)-α increased in the region of the infarction one week after occlusion and declined gradually to return to baseline values at 20 weeks. Moderate upregulation of the expression of cytokines was also seen in the noninfarcted region one week after infarction; however, in contrast to the infarcted regions, cytokines remained significantly increased throughout the entire study. Noninfarcted region cytokine concentrations correlated with the end diastolic diameter of the left ventricle. IL-1β expression was reported as the most prominent. The concentration of IL-1β was shown to correlate well with increased collagen deposition in the noninfarcted myocardium in chronic stages of myocardial infarction [27]. Timely reperfusion helps to limit tissue damage in myocardial infarction. However, reperfusion itself also induces inflammation; therefore, therapeutic manipulation in a clinical setting is challenging [18].

### 2.6. Myocarditis

#### 2.6.1. Viral myocarditis

Almost all viruses might cause myocarditis. Enteroviruses such as coxsackievirus, echovirus, and poliovirus and influenza virus possibly cause myocyte necrosis and acute myocarditis. In severely injured cases, cardiac fibrosis replaces myocyte loss, and cardiac dysfunction persists. Remaining myocytes develop hypertrophy to compensate decreased cardiac function [28]. In contrast, hepatitis C virus (HCV) usually causes chronic inflammation and cardiomyopathies [28]. HCV infects monocytes and macrophages and causes persistent inflammation; this inflammatory reaction may trigger myocyte necrosis, fibrosis, and myocyte hypertrophy [29]. Recently, SARS-CoV2 has been reported to cause myocarditis and cardiovascular diseases [30,31,32]. The exact mechanisms of organ injuries by SARS-CoV2 remain to be clarified, but several mechanisms are suggested [32]. Investigation of SARS-CoV-2 pathogenesis is important to develop preventive and therapeutic approaches for COVID-19.

#### 2.6.2. Autoimmune myocarditis

Autoimmunity, the immune response to self-antigens, can induce myocarditis. We showed that disruption of an inhibitory receptor protein, programmed cell death protein 1 (PD-1), which is expressed on T cells, led to lethal myocarditis in studies with PD-1 knockout mice [33,34]. PD-1 ligands are also expressed in tumor microenvironments [35,36]. PD-1/PD-1 ligand pathway inhibitors exhibit antitumor activity as well as promote T cell activation and are used in cancer treatment. Recent case reports have been published of lethal myocarditis in patients treated with immune checkpoint inhibitors, including an anti-PD-1 drug [37]. These reports highlight that maintenance of immune tolerance is vital to prevent heart autoimmunity. They also demonstrate the importance of vigilant monitoring of cardiac function in patients treated with immune checkpoint inhibitors [19]. Another recent development is reports of increased cases of myocarditis and pericarditis after mRNA COVID-19 vaccination, particularly in teens and young adults [38,39]. Although any possible pathogenetic mechanisms of the COVID-19 mRNA vaccines related to myocarditis remain to be clarified, autoimmunity should be considered to play an important role.

### 2.7. Heart failure

Heart failure had long been considered to be a disease of the heart muscle resulting from chronic activation of the neurohormonal and sympathetic systems. Early clinical reports dating back to the 1950s on an association of C-reactive protein (CRP) with different etiologies of heart failure began to suggest inflammation as an important component to the condition. Following these findings, larger studies conducted in the cytokine era also demonstrated an elevation of inflammatory cytokines in heart failure [19,23]. Subsequent reports indicating an elevation of blood TNF-α and cardiac cachexia have since attracted further attention to the link between cytokines and heart disease. Our work has demonstrated that blood levels of IL-1α, IL-1β, and TNF-α are commonly elevated in myocarditis and, further, that the level of TNF-α is also frequently high in dilated or hypertrophic cardiomyopathy [40]. In addition to the cytokines themselves, blood levels of soluble TNF-α receptor, IL-2 receptor, IL-1 receptor antagonists, and IL-18 have also been shown to be elevated in cardiomyopathy and heart failure patients [23]. Furthermore, elevated blood levels of chemokines and macrophage chemotactic factors, MCP-1, RANTES, and macrophage inflammatory protein-1α have also been reported in heart failure patients [23].

Mast cells detected in human myocardial tissue have been connected with cardiovascular diseases, including the association of higher numbers of mast cells with heart failure [23]. In a previous study conducted in a murine model of systolic pressure overload, our group showed that a mutation at the W/c-kit locus significantly prevented heart failure [41]. These findings indicate that mast cells or a direct c-kit/stem cell factor interaction contributes to the development and progression of heart failure. Lastly, our research has also shown evidence suggesting viral infections may contribute to mast cell activation in the pathophysiology of heart disease as was indicated by the upregulated genetic expression of mast cell chymase and tryptase from the acute stage to heart failure stage [42].

## 3. New biomarkers of inflammation: Immunoglobulin free light chains (FLCs)

The early stages of the inflammatory response are marked by the migration of immune cells to the site of tissue damage. This migration is facilitated by soluble mediators such as cytokines, chemokines, and acute-phase proteins [43]. Chronic inflammation is known to play a significant role in various pathologies, including conditions of aging, cancer, autoimmune diseases, asthma, osteoarthritis, diabetes, atherosclerosis, and cardiovascular diseases [43]. Changes in hematology dynamics, complement factors, acute-phase proteins, and cytokines are common and easily measured in most all inflammatory conditions; however, taken individually, such biomarkers have yet to be strongly associated with specific pathologic events. While these biomarkers are sensitive indicators of inflammation in general, they lack the specificity to identify the offending cause [43].

Elevated CRP levels have been observed in patients with accelerated atherosclerosis, enhanced risk of myocardial infarction, stroke, and vascular disease. CRP might also directly participate in or mediate cytokine-linked plaque instability. It has been shown to be a long-term marker for unexpected sudden cardiac death, serves as a marker of inflammation, and predicts risk of adverse cardiovascular events [44,45,46]. In our study, CRP and IL-6 were increased in patients with acute left heart decompensation in the absence of infection or coronary events. The elevated levels returned toward normal as the symptoms of heart failure resolved. There was significant correlation between peak CRP and peak IL-6 levels [47].

### 3.1. FLCs as new biomarkers of chronic inflammation

**Figure 2:**
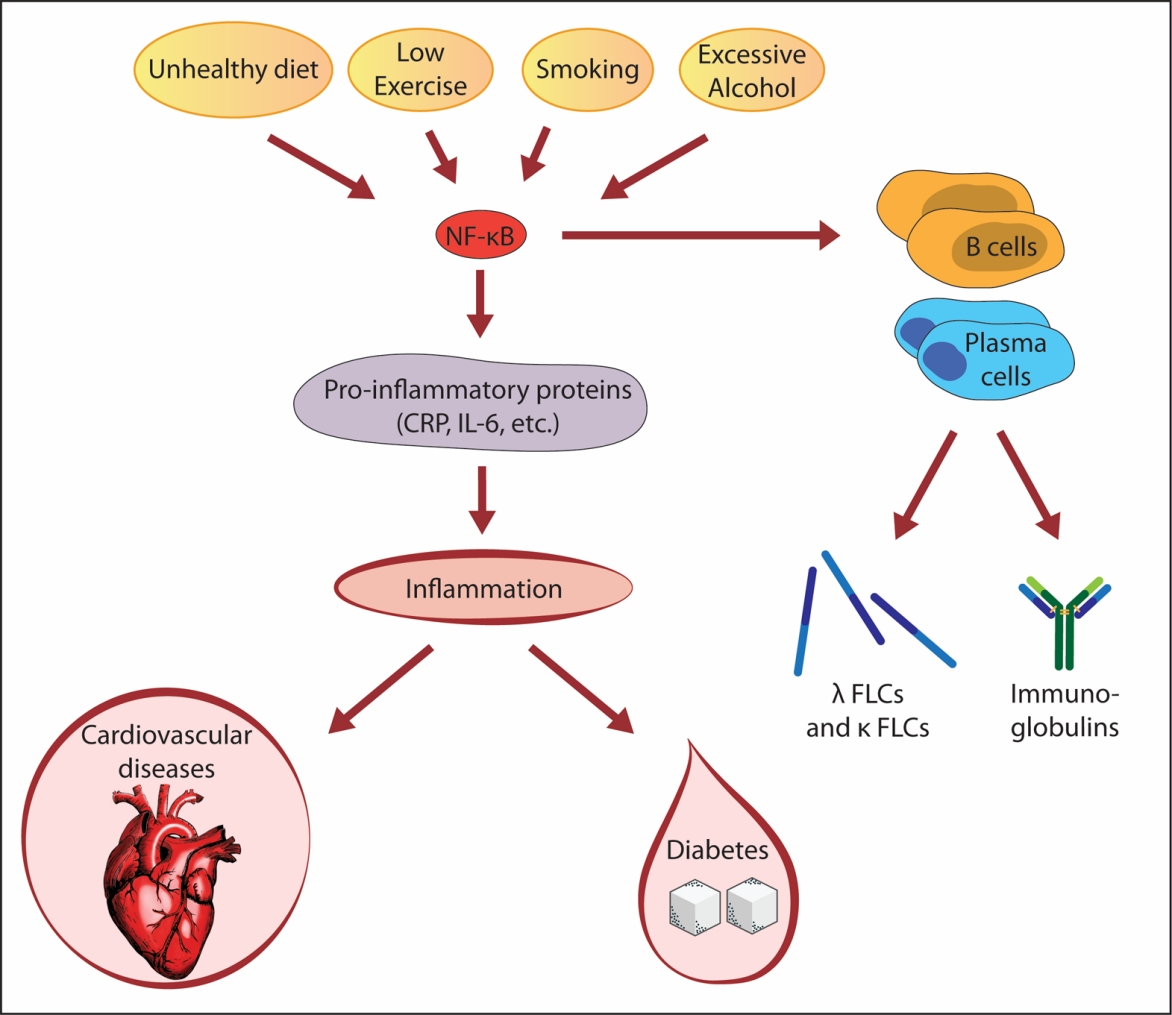
Immunoglobulin free light chains (FLCs) as biomarkers of inflammatory diseases. Risk factors of noncommunicable diseases (NCDs) such as CVDs and diabetes activate nuclear factor kappa B (NF-κB), which regulates the transcription of immunoglobulin free light chain κ in the immunoglobulin-producing B cells and plasma cells and the production of many inflammatory molecules, leading to inflammation. Thus, FLCs were proposed to be biomarkers of NF-κB activation and inflammation.

Serum free light chains (FLCs) are synthesized de novo and secreted into circulation by B cells and plasma cells. As FLCs emerge as an excess by-product of antibody synthesis by B cells and plasma cells, elevated FLCs have been proposed to be a biomarker of B cell activity in many inflammatory and autoimmune conditions (Figure 2) [48]. Polyclonal FLCs are reported to be a predictor of mortality in the general population, measured by the sum of κ and λ concentrations [49]. Increased κ levels occurred in rheumatic diseases, and the κ/λ ratio was higher than in healthy blood donors [50]. FLCs in inflammatory and autoimmune diseases correlate with disease activity, suggesting their role as potential therapeutic targets in such conditions [48,50].

HCV infection can induce insulin resistance and cause diabetes [29]. High concentrations of FLC κ have been observed in HCV-positive patients, and an alteration in the κ/λ ratio is positively correlated with an increasing HCV-related lymphoproliferative disorder severity [51]. Furthermore, it has been suggested that the κ/λ ratio may be useful in the evaluation of therapeutic efficacy [52].

### 3.2. FLCs as markers of heart failure and myocarditis

Our group observed FLCs to be increased in a mouse model of heart failure due to viral myocarditis [53]. In more recent research in heart failure patients, we observed that circulating FLC λ were increased while the κ/λ ratio was decreased in sera from patients with heart failure with myocarditis compared to a group of healthy controls. These findings demonstrated that the FLC λ and κ/λ ratio together showed a good diagnostic ability for the identification of myocarditis. Further, the FLC κ/λ ratio also demonstrated to be an independent prognostic factor for overall patient survival [54]. Most recently, our work involving COVID-19 myocarditis patients [31] also found FLC λ was elevated in 80% of patients. FLCs were seen to be more frequently increased than troponin, creatine kinase, or N-terminal prohormone of brain natriuretic peptide (NT-proBNP), suggesting that FLCs may be a sensitive biomarker of COVID-19 myocarditis. The answer to the question as to why specific activation of FLC λ expression would occur in this situation is yet to be determined, but it does suggest that the cells producing FLC λ, namely clones of B cells and plasma cells, are specifically activated in myocarditis [32]. NF-κB may not exercise control of the production of FLC κ and λ in the same manner, leaving open the possibility that FLC κ and λ are regulated differently [32]. Based on the findings of our research, we propose that NF-κB may present a new target for anti-inflammatory therapies in myocarditis with elevated FLCs and, further, that FLCs may serve as an alternate endpoint for the treatment of myocarditis.

### 3.3. FLCs as markers of atrial fibrillation

Atrial fibrillation is the most common arrhythmia that is an important cause of stroke. It is important to develop a method to detect early or asymptomatic atrial fibrillation. Abnormal atrial histology compatible with a diagnosis of myocarditis was uniformly found in patients with lone atrial fibrillation. Patients with atrial fibrillation exhibited a higher concentration of cytokines, higher NF-κB activity, and more severe lymphomonocyte infiltration than those in sinus rhythm. These observations imply local immunologic inflammatory responses in the atria in atrial fibrillation [55]. The concentrations of circulating FLC κ and λ in patients with lone atrial fibrillation were significantly different from the healthy volunteers group. The area under the curve of the receiver operating characteristic curve (ROC) analysis showed that FLC κ and λ were helpful in differentiating atrial fibrillation from healthy volunteers and that the cutoff value of FLC κ or λ may be beneficial to distinguish both groups [55]. The mechanism by which FLCs cause atrial fibrillation remains to be clarified, but the inflammation associated with FLCs directly induces atrial fibrillation or FLCs might cause a change in membrane fluidity, which, in turn, could alter ion channel function [55].

### 3.4. FLCs as markers of diabetes, a risk factor of CVDs

Chronic inflammation is increasingly recognized as a significant factor in the pathogenesis of non-insulin-dependent diabetes mellitus and associated negative sequalae. Diabetes patients commonly present with elevated levels of serum IL-1β, IL-6, and CRP, indicating a clear inflammatory component to their condition [56]. In our recently published study, we observed FLC κ was decreased, λ was elevated, and the κ/λ ratio was decreased in patients with diabetes compared to the healthy controls [57]. The reason why FLC λ appears to increase in diabetes is still to be determined; however, these findings strongly suggest that the clones of the B cells and plasma cells that produce FLC λ are being specifically activated in diabetes. The area under the curve in ROC analysis of FLC λ and the κ/λ ratio is larger compared with healthy controls, and sensitivity and specificity for the diagnosis are very high. Sensitivity and specificity for the diagnosis of diabetes by FLC λ and the κ/λ ratio are higher than those of glycated hemoglobin A1c (HbA1c). Urine λ and the κ/λ ratio were well correlated with those of sera, suggesting that urine FLCs could be a suitable and noninvasive biomarker of diabetes (unpublished observation). Since HbA1c cannot be measured in urine, FLCs would be more beneficial biomarkers of diabetes than HbA1c. Since FLCs are considered as a marker of inflammation/immune activation, their presence in diabetes confirms the inflammatory/immune character of this disease.

## 4. FLCs as a screening test and evaluation of the interventions for CVDs and NCDs

Determination of FLCs is suggested to be suitable as an initial health screening in the general population. When abnormalities of FLCs are found, secondary tests should be performed and followed up: BNP/NT-proBNP for heart failure, HbA1c for diabetes, and electrocardiograms for atrial fibrillation. Follow-up measurements of FLCs are useful to evaluate the effects of, e.g., diet, lifestyle changes, or drug therapy.

**Figure 3:**
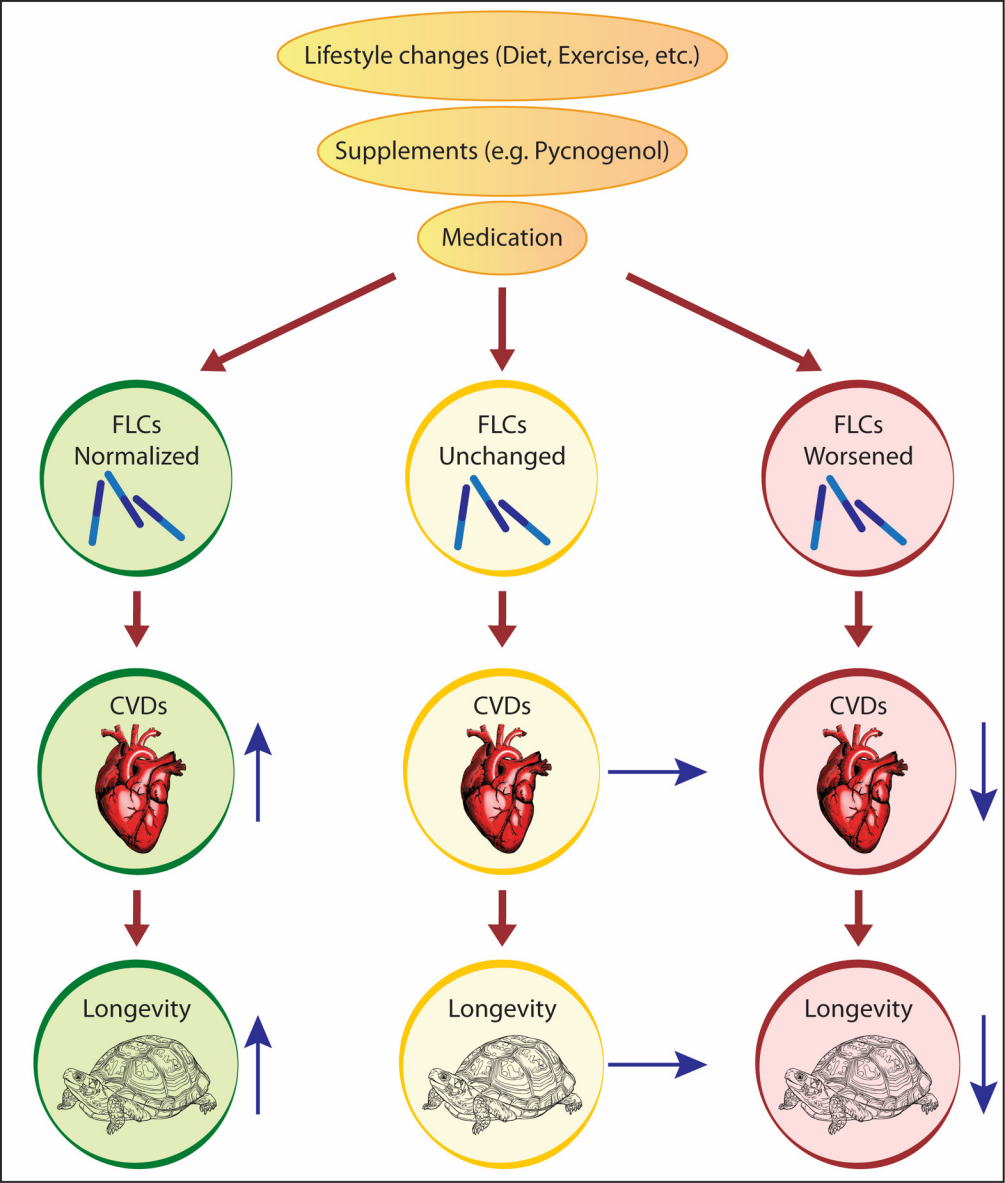
FLCs as possible predictive biomarkers of longevity. Sequential measurements of FLCs before and after the intervention of lifestyle changes (diet, exercise, etc.) or intake of supplements or drugs could predict their benefit for the longevity.

So far, there are no reliable biomarkers to predict longevity. When FLCs are measured sequentially, it might offer the chance to determine an optimal individual strategy, such as changes of lifestyles (diet, exercise, etc.) or the intake of supplements or drugs. If FLCs normalize, the lifestyle changes would be beneficial for an individual, but if FLCs further deteriorate, the changes would not be beneficial for health and longevity (Figure 3).

## 5. Anti-inflammatory strategies for the prevention and management of CVDs

### 5.1. Anti-inflammatory diets

Unhealthy diets focused on refined sugars, fried foods, sodas, lard, and processed meat strongly contribute to the pro-inflammatory effect of the diet [8]. The opposite has also been clearly shown, with research on healthy plant-based diets showing their importance in the prevention of CVDs [58]. The most well-researched and highly reputed diets include the DASH (Dietary Approach to Stop Hypertension), Mediterranean, and Flexitarian diets [59]. The accumulation of scientific evidence, mainly in the form of well-designed cohort studies but also from a few randomized controlled trials (RCTs), has helped to increase awareness and acceptance of these healthy dietary patterns. RCTs have been conducted to assess the effects of different diets on critical health conditions with a heavy focus on the incidence of CVDs and mortality but also on neurodegenerative diseases, diabetes, and cancer as well as all-cause mortality [60]. In their paper published in 2020, Li and coworkers [8] recommended the consumption of antioxidant-rich foods, starting with green leafy vegetables (kale, spinaches, cabbage watercress, Swiss chard, arugula, endive), yellow vegetables (pumpkin, yellow peppers, beans, carrots), whole grains (wheat, oat, rye, buck wheat, millet), and beverages such as coffee, tea, and wine. Results of a RCT conducted by Cofan and coworkers [61] suggest walnuts should be added to this list based on their high polyphenol content and nutrient profile. Other anti-inflammatory foods that should be considered as part of a health-promoting diet also include extra-virgin olive oil, fatty fish, and tomatoes as well as colorful high-fiber and polyphenol-rich fruits, like blueberries, pomegranate, orange, cherries, strawberry, apples, and pears [62]. Foods focused on in these healthy diets share common factors such as being nutrient dense and rich sources of anti-inflammatory compounds such as vitamins, carotenoids, polyphenols, fiber, and long-chain omega-3 fatty acids [63]. Current research suggests that polyphenols are the most important food-based bioactive compounds in terms of anti-inflammatory activity. A large number of compounds, including flavonoids such as anthocyanins (present mainly in berries and wine), flavanols (catechins) present in cocoa and tea, and flavonols (quercetin) in onions, among others, have shown significant anti-inflammatory properties. Other nonflavonoid, phenolic compounds that demonstrate anti-inflammatory actions are also extensively present in nature in the form of phenolic acids, lignans, stilbenes, and others [64].

Antioxidant activity continues to be a main focus in explaining the health-promoting actions of foods. Many foods, particularly those of high polyphenol content, exhibit potent antioxidant capacity in *in vitro* studies. It is important to note that many questions regarding the antioxidant properties of food and whether or not *in vitro* measurements have any usefulness in explaining their effectiveness in the human body remain to be answered [65]. The most popular test is the oxygen radical absorbance capacity (ORAC) assay, which measures the antioxidant capacity of substances *in vitro*. A large variety of food compounds have been tested using the ORAC assay, and ORAC values continue to be routinely misused by marketers of health foods and dietary supplements in the promotion of their products. Issues related to the absorption and metabolism as well as antioxidant capacity *in vivo* of most food compounds still require further research. Use of *in vitro* data can be misleading to the consumer because the data related to the antioxidant capacity of food compounds generated in a laboratory by *in vitro* methods cannot be extrapolated to *in vivo* effects upon consumption. Clinical trials to test benefits of dietary antioxidants have produced mixed results. As the research progresses, it is becoming clear that antioxidant molecules in foods have a wide range of functions, many of which are unrelated to the capacity to neutralize reactive oxygen species [66].

### 5.2. Anti-inflammatory effects of the French pine bark extract of Pycnogenol^®^

Pycnogenol® (PYC), a natural plant extract derived by a patented, standardized extraction process from the bark of the French maritime pine (*Pinus pinaster*, subsp. *atlantica*), is a highly concentrated source of flavanols as well as phenolic and cinnamic acids [67]. Anti-inflammatory *in vitro* effects of PYC have been confirmed in *ex vivo* studies using human plasma following intake of PYC in which PYC intake was shown to induce decreased gene expression of COX-2 and inducible nitric oxide synthase (iNOS) enzymes as well as decrease activation of NF-κB and activity of COX-1, COX-2 and phospholipase A2, and decrease the release of elastase [68,69]. PYC inhibited NF-κB activation and decreased induction of TNF-α, intracellular adhesion molecule-1 (ICAM-1) and vascular cell adhesion molecule-1 (VCAM-1) gene expression [70].

#### 5.2.1. Antiviral effects of PYC on viral myocarditis in animals

**Figure 4:**
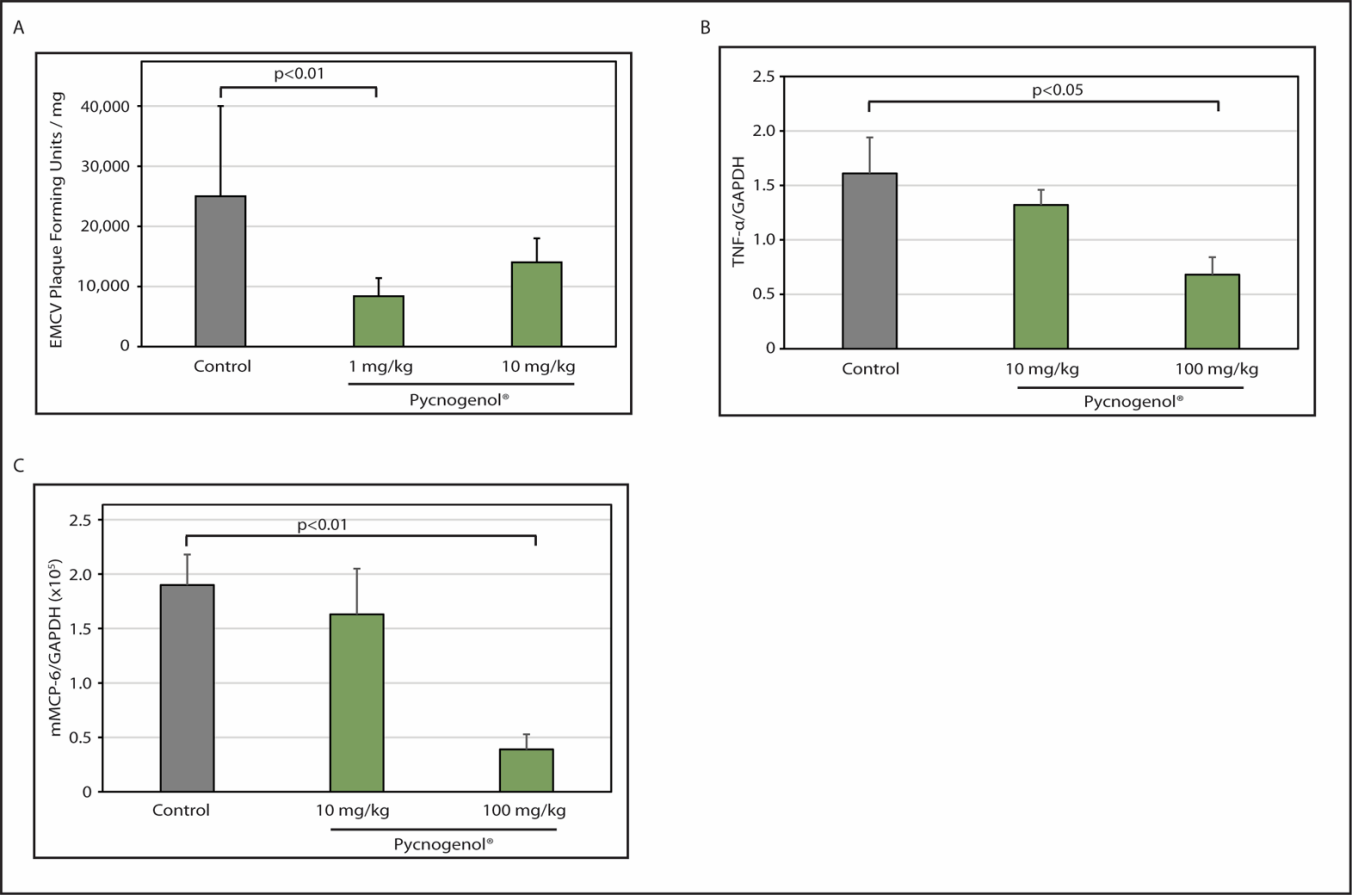
Antiviral effects of Pycnogenol (PYC) on encephalomyocarditis viral (EMCV) infection in mice. **(A)** The amount of plaque forming units in the hearts of mice after PYC oral administration (1 mg/kg and 10 mg/kg) and in control mice. **(B)** Expression levels of plasma pro-inflammatory cytokine TNF-α in control mice and in mice after PYC oral administration (10 mg/kg and 100 mg/kg). **(C)** Expression levels of plasma mast cell–related tryptase mMCP-6 in control mice and in mice after PYC oral administration (10 mg/kg and 100 mg/kg). Reproduced from Matsumori et al. [70] with permission of Elsevier.

Our group conducted a study using PYC to investigate the anti-inflammatory properties in a mouse model of viral myocarditis. Results showed that PYC inhibited the replication of the encephalomyocarditis virus (EMCV) both *in vitro* and *in vivo* in animals and improved inflammation and preserved myocardial tissue by decreasing necrosis [70]. Administration of PYC significantly inhibited the gene expression of pro-inflammatory cytokines while also inhibiting expressions of mast cell–related tryptase and stem cell factor (Figure 4). In related research, our group has reported that inhibiting activation of NF-κB and iNOS provided significant beneficial effects in EMCV myocarditis. As PYC displays similar inhibitory activity against NF-κB and iNOS, this may be a part of the mechanisms by which the extract improved EMCV viral myocarditis.

#### 5.2.2 Antiviral effects of PYC on hepatitis C virus (HCV)

PYC treatment showed antiviral effects against the hepatitis C virus (HCV) and synergistic effects with interferon-α or ribavirin *in vitro* [71]. PYC worked additively with the antiviral preparation, telaprevir, to lower levels of HCV RNA in wild-type HCV replicon cells while showing no cytotoxic effects. Further, PYC was shown to inhibit viral replication in telaprevir-resistant replicon cells as well. Investigations into the efficacy of PYC extract fractions revealed that the extract as a whole PYC showed significantly higher antiviral activity than its individual components, procyanidin and taxifolin. *In vivo* investigations in HCV-infected chimeric mice revealed that PYC again inhibited HCV replication. Further, the extract showed a synergistic antiviral effect with interferon-α. Lastly, the addition of PYC to HCV replicon cell lines resulted in a dose-dependent reduction of reactive oxygen species [71].

#### 5.2.3. Antiviral effects of PYC on hepatitis B virus (HBV) in humans

**Figure 5:**
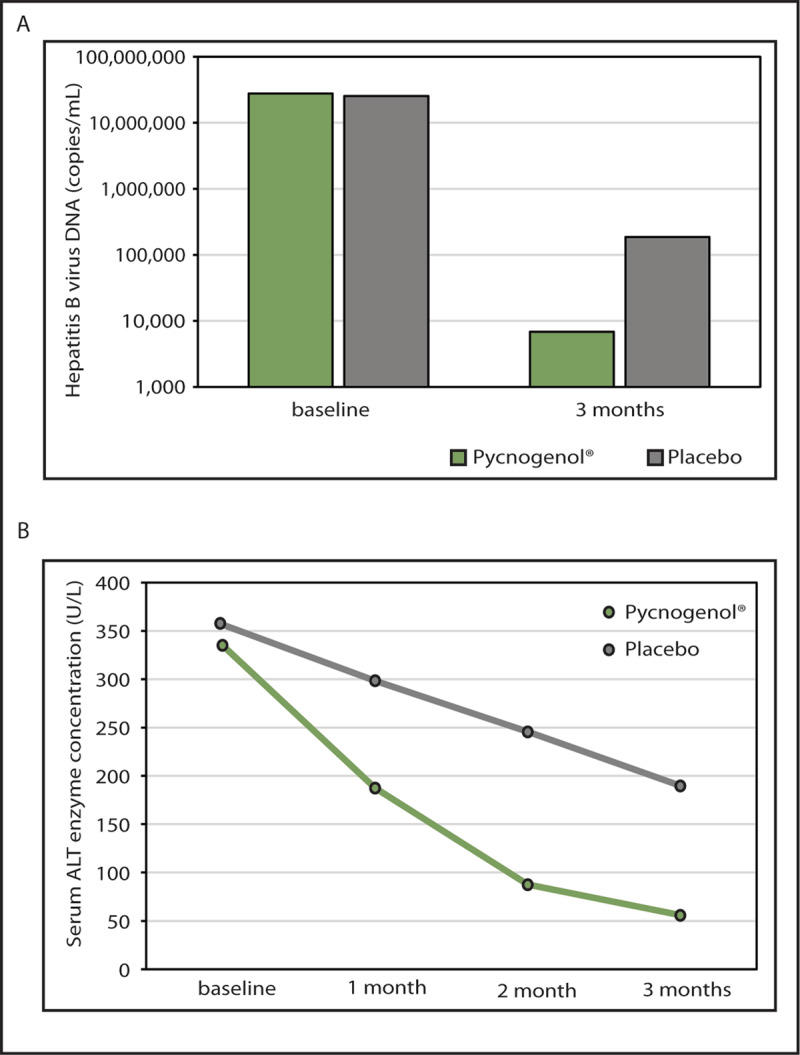
The effect of PYC on hepatitis B virus in humans. **(A)** Copies of hepatitis B virus DNA per mL in the blood of patients after PYC or placebo administration. **(B)** Hepatic serum ALT enzyme concentration (U/L) in patients after PYC or placebo administration.

We performed a double-blind placebo-controlled study involving patients with hepatitis B virus (HBV) infection. PYC 200 mg or a placebo was given daily over 12 weeks. In the PYC group, circulating HBV was reduced and hepatic function was improved after administration of PYC [72,73] (Figure 5).

**Figure 6:**
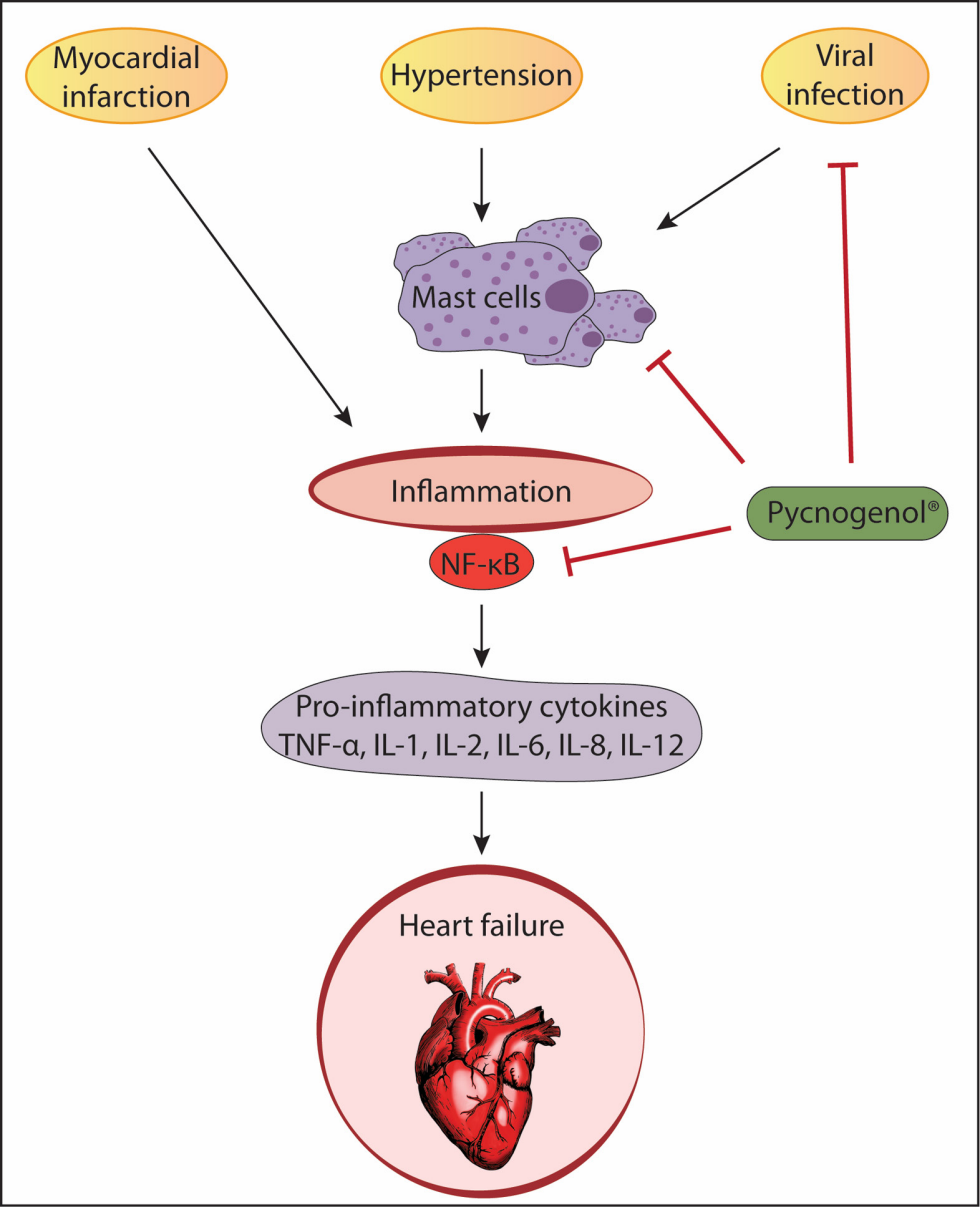
Potential mechanisms of PYC on heart failure. PYC may prevent heart failure by inhibiting inflammation by multiple pathways such as antiviral effects and inhibiting activation of NF-κB and mast cells.

Figure 6 summarizes the anti-inflammatory effects of PYC. PYC may prevent heart failure and CVDs by inhibiting inflammation by multiple pathways.

## Conclusions

The role of inflammation in the development and progression of CVDs is important. Biomarkers reflecting inflammation of CVDs are important for patient assessment. FLCs are novel biomarkers of inflammation, and their levels have been associated with overall mortality in a general population and with cardiovascular outcomes. Thus, FLCs are promising biomarkers for the evaluation of interventions for improved longevity. PYC has significant anti-inflammatory effects against various diseases as well as antiviral effects, making it promising for the prevention and management of various CVDs.
